# 10 years of CEMARA database in the AnDDI-Rares network: a unique resource facilitating research and epidemiology in developmental disorders in France

**DOI:** 10.1186/s13023-021-01957-4

**Published:** 2021-08-04

**Authors:** Claude Messiaen, Caroline Racine, Ahlem Khatim, Louis Soussand, Sylvie Odent, Didier Lacombe, Sylvie Manouvrier, Patrick Edery, Sabine Sigaudy, David Geneviève, Christel Thauvin-Robinet, Laurent Pasquier, Florence Petit, Massimiliano Rossi, Marjolaine Willems, Tania Attié-Bitach, Pierre-Henry Roux-Levy, Laurent Demougeot, Lilia Ben Slama, Paul Landais, the AnDDI-Rares network, Bruno Leheup, Martine Doco-Fenzy, Céline Poirsier, Marta Spodenkiewicz, Lola Lissy, Audrey Lannoy, Elise Shaefer, Salima El Chehadeh, Jeanne Amiel, Cyril Mignot, Judith Melki, Sandra Whalen, Marilyn Irène Lackmy, Benoit Funalot, Gilles Morin, Marion Gérard, Nicolas Gruchy, Arnaud Molin, Annick Toutain, Stéphanie Arpin, Sophie Blesson, Médéric Jeanne, Bertrand Isidor, Marie Vincent, Mathilde Nizon, Sandra Mercier, Dominique Bonneau, Estelle Colin, Alban Ziegler, Séverine Audebert-Bellanger, Radka Stoeva, Florence Demurger, Julien Thevenon, Christine Francannet, Baptiste Troude, Isabelle Perthus, Damien Haye, Patrick Collignon, Brigitte Gilbert-Dussardier, Frédéric Bilan, Mattieu Egloff, Gwenaël Le Guyader, Pascaline Letard, Elisabeth Sarrazin, Anna-Gaëlle Giguet-Valard, Léna Damaj, Mélanie Fradin, Alinoe Lavillaureix, Nolwenn Jean-Marçais, Godelieve Morel, Chloé Quelin, Sophie Naudion, Marine Legendre, Julien Van-Gils, Caroline Rooryck-Thambo, Odile Boute, Anne Dieux, Catherine Vincent-Delorme, Jamal Ghoumid, Clémence Vanlerberghe, Roseline Caumes, Cindy Colson, Luisa Marsili, Antoine Wyrebski, Laurence Bellengier, Françoise Houdayer, Audrey Putoux, Tiffany Busa, Florence Riccardi, Chantal Missirian, Patricia Blanchet, Christine Coubes, Emmanuelle Haquet, Lucile Pinson, Jacques Puechberty, Constance Wells, Yline Capri, Laurence Perrin, Sandrine Passemard, Lyse Ruand, Sophie Nambot, Julian Delanne, Sébastien Moutton, Arthur Sorlin, Daphné Lehalle, Aurore Garde, Anne-Sophie Jannot, Christine Binquet, Arnaud Sandrin, Alain Verloes, Laurence Faivre

**Affiliations:** 1 Banque Nationale de Données Maladies Rares, DSI-WIND, APHP, Paris, France; 2 Centre de Référence Anomalies du Développement et Syndromes Malformatifs, CHU de Dijon, Dijon, France; 3 Centre de Référence Anomalies du Développement et Syndromes Malformatifs, Hôpital Sud, CHU de Rennes, Rennes, France; 4 Centre de Référence Anomalies du Développement et Syndromes Malformatifs, CHU Bordeaux, et INSERM U1211, Bordeaux, France; 5 Centre de Référence Anomalies du Développement et Syndromes Malformatifs, CHU de Lille, EA 7364 RADEME Maladies Rares du Développement et du Métabolisme, Université Lille, Lille, France; 6 Centre de Référence Anomalies du Développement et Syndromes Malformatifs, Hôpital Femme-Mère-Enfant Hospices Civils de Lyon, Bron, France; 7 Centre de Référence Anomalies du Développement et Syndromes Malformatifs, Département de Génétique Médicale, CHU de Marseille - Hôpital de La Timone, Marseille, France; 8 Centre de Référence Anomalies du Développement et Syndromes Malformatifs, CHU Montpellier, Montpellier, France; 9 Filière AnDDI-Rares, CHU Dijon, Dijon, France; 10 INSERM UMR1231 et FHU TRANSLAD, Université de Bourgogne, Dijon, France; 11 Hôpital Necker-Enfants Malades, Paris, France; 12 Service de Biostatistique, Epidémiologie, Santé Publique et d’Information Médicale, CHU de Nîmes, Faculté de Médecine Montpellier Nîmes, Nîmes, France; 13 Inserm, CIC1432, module épidémiologie clinique, Dijon, France; 14 CHU Dijon-Bourgogne, Centre d’Investigation Clinique, Epidémiologie Clinique/Essais Cliniques, Dijon, France; 15 Centre de Référence Anomalies du Développement et Syndromes Malformatifs, AP-HP-Nord-Université de Paris, Hôpital Robert Debré, Department of Medical Genetics and INSERM UMR 1141, Paris, France; 16 AP-HP. Centre - Université de Paris, Paris, France; 17 Centre de Génétique et Centre de Référence Anomalies du Développement et Syndromes Malformatifs, Filière AnDDI-Rares, Hôpital D’Enfants, CHU Dijon, 14 rue Gaffarel, Dijon, France; 18 Hôpital d’Enfants CHU de Nancy - Hôpitaux de Brabois, Vandœuvre-lès-Nancy, France; 19 CHU de Reims - Hôpital Maison Blanche, Reims, France; 20 CHU de Strasbourg - Hôpital de Hautepierre, Strasbourg, France; 21 CHU Paris - Hôpital Necker-Enfants Malades, Paris, France; 22 Service de Génétique Clinique et Médicale CHU Paris-GH La Pitié Salpêtrière-Charles Foix - Hôpital Pitié-Salpêtrière, Paris, France; 23 GHU Paris-Sud - Hôpital de Bicêtre, Le Kremlin-Bicêtre, France; 24 CHU Hôpital d’Enfants Armand-Trousseau, Paris, France; 25 CHU de Pointe à Pitre, Guadeloupe, France; 26 Centre Hospitalier Intercommunal de Créteil, Créteil, France; 27 Amiens-Picardie - Site Sud, Amiens, France; 28 CHU de Caen - Hôpital Clémenceau, Caen, France; 29 Pôle Gynécologie Obstétrique - Médecine Foetale - Reproduction et Génétique CHRU de Tours - Hôpital Bretonneau, Tours, France; 30 Service de Génétique Médicale CHU de Nantes - Hôtel Dieu, Nantes, France; 31 CHU d’Angers, Angers, France; 32 Pôle Femme-Mère-Enfant CHRU de Brest - Hôpital Morvan, Brest, France; 33 Pôle de Biopathologie, UF 3162 Centre Hospitalier du Mans, Le Mans, France; 34 CHBA Centre hospitalier Bretagne Altantique – CH Chubert, Vannes, France; 35 CHU de Grenoble site Nord – Hôpital Couple-Enfant, La Tronche, France; 36 Pôle de pédiatrie CHU de Clermont-Ferrand – Hôpital d’Estaing, Clermont-Ferrand, France; 37 CHU de Nice – Hôpital l’Archet 2, Nice, France; 38 Service de génétique médicale, CHI de Toulon, Toulon, France; 39 CHU de Poitiers, Poitiers, France; 40 CHU de Fort de France, Fort-de-France, France; 41 CHU de Rennes – Hôpital Sud, Rennes, France; 42 CHU de Bordeaux, Bordeaux, France; 43 CHRU de Lille, Lille, France; 44 Hôpital Femme-Mère-Enfant Hospices Civils de Lyon, Lyon, France; 45 CHU de Marseille - Hôpital de La Timone, Marseille, France; 46 CHU de Montpellier, Montpellier, France; 47 AP-HP-Nord-Université de Paris – Hôpital Robert Debré, Paris, France; 48 CHU de Dijon, CHU de Dijon, France

**Keywords:** Rare disease, Developmental disorders, Data warehouse, Epidemiology

## Abstract

**Background:**

In France, the Ministry of Health has implemented a comprehensive program for rare diseases (RD) that includes an epidemiological program as well as the establishment of expert centers for the clinical care of patients with RD. Since 2007, most of these centers have entered the data for patients with developmental disorders into the CEMARA population-based registry, a national online data repository for all rare diseases. Through the CEMARA web portal, descriptive demographic data, clinical data, and the chronology of medical follow-up can be obtained for each center. We address the interest and ongoing challenges of this national data collection system 10 years after its implementation.

**Methods:**

Since 2007, clinicians and researchers have reported the “minimum dataset (MDS)” for each patient presenting to their expert center. We retrospectively analyzed administrative data, demographic data, care organization and diagnoses.

**Results:**

Over 10 years, 228,243 RD patients (including healthy carriers and family members for whom experts denied any suspicion of RD) have visited an expert center. Among them, 167,361 were patients affected by a RD (median age 11 years, 54% children, 46% adults, with a balanced sex ratio), and 60,882 were unaffected relatives (median age 37 years). The majority of patients (87%) were seen no more than once a year, and 52% of visits were for a diagnostic procedure. Among the 2,869 recorded rare disorders, 1,907 (66.5%) were recorded in less than 10 patients, 802 (28%) in 10 to 100 patients, 149 (5.2%) in 100 to 1,000 patients, and 11 (0.4%) in > 1,000 patients. Overall, 45.6% of individuals had no diagnosis and 6.7% had an uncertain diagnosis. Children were mainly referred by their pediatrician (46%; n = 55,755 among the 121,136 total children referrals) and adults by a medical specialist (34%; n = 14,053 among the 41,564 total adult referrals). Given the geographical coverage of the centers, the median distance from the patient’s home was 25.1 km (IQR = 6.3 km-64.2 km).

**Conclusions:**

CEMARA provides unprecedented support for epidemiological, clinical and therapeutic studies in the field of RD. Researchers can benefit from the national scope of CEMARA data, but also focus on specific diseases or patient subgroups. While this endeavor has been a major collective effort among French RD experts to gather large-scale data into a single database, it provides tremendous potential to improve patient care.

**Supplementary Information:**

The online version contains supplementary material available at 10.1186/s13023-021-01957-4.

## Background

Rare diseases (RD) are diseases with a prevalence inferior to one in 2000 in the general population. Though rare, they are a major public health concern since they are collectively common, and 2–3% of births and 7–8% of adults are or will be affected by an RD [1]. More than three million French people and about 25 million Europeans are affected by one of the 7,000 currently recognized RD. In half of all cases, RD affect children under 5 years, and they are responsible for 10% of deaths in children aged 1 to 5 years [1]. Eighty percent of RDs are of genetic origin. Most often, they are severe chronic diseases, and they can also be progressive. They considerably affect the quality of life of affected patients, causing motor, sensory or intellectual deficits in 50% of cases, and total dependency in 9% of cases [1]. There is a crucial lack of treatment for RD, since only 5% of these disorders have an available treatment [2]. For these reasons, three French national plans (Plan National Maladies Rares or PNMR) have been successively established for RD since 2004, enabling France to play a leading role in the field of RD in Europe [3]. The first PNMR structured a national network of 131 multidisciplinary reference centers for RD (RCRD) and more than 500 centers of expertise for RD (CERD), which was then revised with the 3rd PNMR, resulting in a total of 387 RCRD and 1,800 CERD. The RCRD form a network of national excellence centers with extensive geographical coverage. The CERD provide RCRD expertise to local hospitals. This network gives patients the opportunity to access comprehensive clinical work-ups and regular follow-up as close as possible to their homes. The interactions between the RCRD and expert clinical laboratories, research laboratories, patient support groups, and the other various medico-social specialties in the patient care pathways have been structured into 23 thematic networks for RD (each of them encompassing RCRD and CERD for one group of diseases, accredited by the 2nd PNMR) [4]. Their objectives are to optimize the supply of care, improve education and training, and stimulate the development of research and innovation in the field of RD. The way in which patients with RD are managed in France strongly inspired the creation of European Reference Networks by the European Commission [5].

The PNMR have focused on improving knowledge about the epidemiology of RD through the constitution of a dedicated registry collecting information from the rare disease network. In order to fulfill this objective, a population-based registry, called CEMARA, was launched in 2007. It collects epidemiological information about RD and related medical activities from RCRD and CERD on a national level. The goal of CEMARA was to improve the understanding of the burden of disease for rare conditions, to determine the resources needed for healthcare and social services, and to identify patients eligible for natural history studies and clinical trials [6]. A minimum dataset (MDS) [31] has been set up (Additional file 1: Table S1). Physicians and paramedical workers (psychologists, genetic counsellors, and social workers) enter data from the RD centers, and the system allows longitudinal follow-up of individual patients. The French Data Protection Authority authorized CEMARA in 2007. It is compliant with European GDPR regulation. A Scientific Committee has validated the studies issued from the CEMARA data. The CEMARA project has registered 500,000 RD patients from 151 RCRD (out of 387), 412 CERD and recorded over 4000 RD.

Among the 23 accredited health networks, AnDDI-Rares (Anomalies du Développement avec ou sans Déficiences Intellectuelles de causes Rares) is the network of medical genetic services implanted in university hospitals. It focuses on individuals with developmental abnormalities (malformations and intellectual disability (ID)) or not, and works with more than 5000 distinct rare monogenic diseases and a large number of chromosomal abnormalities [7]. These diseases have a prevalence of 3% (about 1.8 million people and 40,000 new cases per year in France). These disorders share common characteristics: (i) an often difficult diagnosis requiring clinical and biological expertise, (ii) a high rate of patients with no diagnosis, (iii) coordinated care relying often on multidisciplinary therapy facilities and special-needs schooling requiring multiple interactions between hospital and non-hospital partners, and (iv) the need for epidemiological, clinical and translational research regarding the natural history and pathophysiology of developmental abnormalities, with a focus on long-awaited therapeutic solutions (often requiring multicenter cohort studies). Initially, according to the first PNMR, the AnDDI-Rares network included 22 constitutive RCRD grouped under the supervision of 8 coordinator RCRD, and 7 CERD. Currently, AnDDI-Rares includes 20 constitutive university hospitals grouped under the coordination of six RCRD (one per large French inter-region), and 29 further CERD (Additional file 2: Figure S1A). Besides the facilities for care and treatment, AnDDI-Rares includes diagnostic laboratories (38 for molecular genetics, 44 for cytogenetics, 48 fetal pathology units), 32 research teams, and over 60 family support groups. The 26 departments forming the AnDDI-Rares RCRD (beneficiary of an operating grant from the French state) have filled out the register since 2007. Participation of the CERD (which does not have a grant) was optional.

Here, to gain knowledge about patients with developmental disorders and their care pathway in France, we studied the cohort of patients followed up in AnDDI-Rares network for developmental disorders, using data from the first 10 years of CEMARA data collection. We then focused on four sub-cohorts of patients diagnosed with four different specific diseases to study their characteristics and follow-up. Lastly, we focused on the sub-cohort of patients with chromosomal anomalies.

## Methods

### Study design

**Fig. 1:**
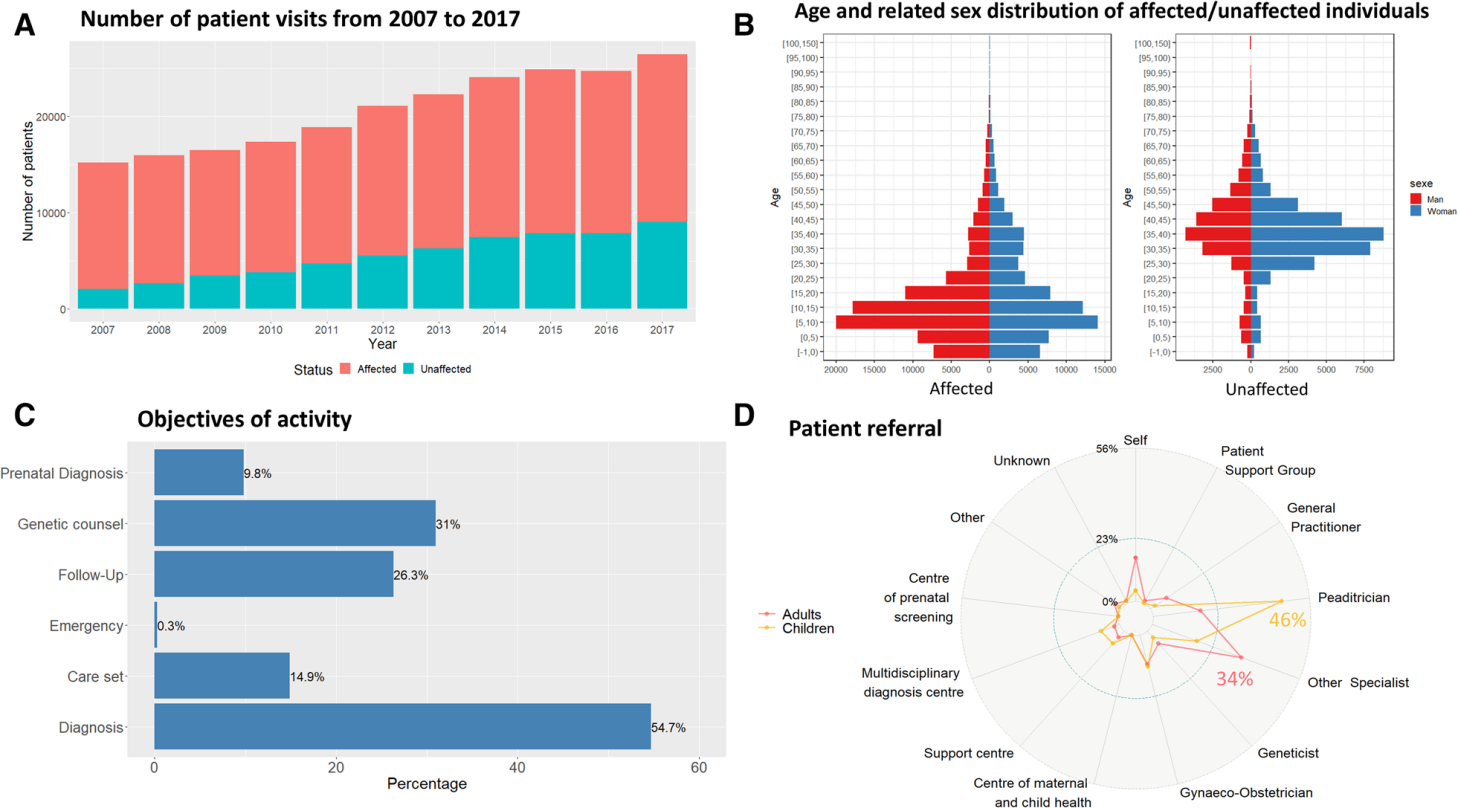
Demographic information, Care pathways. **A** Number of patient visits from 2007 to 2017, comprising affected individuals (red) and non-affected relatives (blue). **B** Age and related sex distribution of affected/unaffected individuals. The median age in affected individuals was 11 years, and the median age in non-affected individuals was 37 years. Within the affected population, 51.6% (n = 86,304) of the affected patients were males and 44.4% females (n = 74,319). **C** Objectives of activity. 55% (n = 208,433) of activities were for diagnosis purposes, 31% (n = 118,244) for genetic counseling, and only 36% (n = 136,286) for follow-up (FU) care, 9.7% (n = 37,397) for prenatal diagnosis, and 0.3% (n = 999) for emergency, taking into account that the same patient can be seen for more than one reason. **D** Patient referral. Patients are mainly referred to a RCRD/CERD by a pediatrician when the index case is a child (n = 55,755; 46%), and by a specialist when the affected patient is an adult (n = 14,053; 34%). For Children: Self: 4% (n = 5242); Patient Support Group: 0% (n = 373); General Practitioner: 2% (n = 2399); Pediatrician: 46% (n = 55,755); Other Specialist: 17% (n = 20,555); Geneticist: 3% (n = 3372); Gynecologist-Obstetrician: 12% (n = 14,778); Centre of maternal and child health: 0% (n = 340); Support center: 6% (n = 7345); Multi-disciplinary diagnosis center: 7% (n = 8599); Prenatal screening centre: 0% (n = 163); Other: 1% (n = 1322); Unknown: 1% (n = 893). For Adults: Self: 16% (n = 6452); Patient Support Group: 1% (n = 324); General Practitioner: 7% (n = 3073); Pediatrician: 17% (n = 7050); Other Specialist: 34% (n = 14,053); Geneticist: 6% (n = 2495); Gynecologist-Obstetrician: 11% (n = 4434); Centre of maternal and child health: 0% (n = 51); Support center: 3% (n = 1219); Multi-disciplinary diagnosis center: 2% (n = 713); Prenatal screening centre: 0% (n = 11); Other: 3% (n = 1186); Unknown: 1% (n = 503)

We performed a cross-sectional cohort study on a population-based cohort. We included all the consultations of all patients with developmental abnormalities seen in a RCRD or a CERD of the AnDDI-Rares network (Fig. 1A) within the 2007–2017 period in France. Tele-expertise or expert opinions on medical files were reported in some cases and therefore included in the study.

### Data collection

An MDS with mandatory and optional information was collected for each patient and visit so that all centers had a common core data set (Additional file 1: Table S1). The following items were mandatory for the MDS: demographics data (birth and death date, sex, residential address) for the index case and family members when necessary, diagnosis, type of visit, and objective of the activity. An identification module ensured that there were no identity doubles using a double-entry prevention function [8].

If necessary, several diagnoses were provided for a given patient. Optional information included antenatal or neonatal data, mode of inheritance, additional keywords for describing atypical signs and symptoms, or for patients presenting with a still unknown diagnosis (Additional file 1: Table S1). For the diagnosis labels of the patient records, a classification was set up with the health professionals corresponding to their needs. The database was linked to Orphanet, which has designed specific thesauri dedicated to RD. A diagnosis was considered as: ‘confirmed’ when the diagnosis was confirmed with a cytogenetic or molecular method or when other criteria were considered sufficient to support the diagnostic confirmation and no additional investigations were deemed necessary; ‘likely’ if the diagnostic hypothesis was likely given the available data, but not all the signs or tests necessary to confirm the diagnosis were available; ‘ongoing’ when the diagnosis was in progress and no examination results had yet come back for this diagnosis; ‘indeterminate’ when the physician could not give an opinion on the diagnosis in the absence or unavailability of diagnostic tests, or due to non-contributory tests. When patients did not have a diagnosis (‘unlabeled’), they could be classified according to a diagnostic category, such as developmental disorder with ID, non-syndromic ID, developmental disorder without ID, malformative syndrome with short stature, syndromic epilepsy. Also, to annotate the diagnosis, the database provided keywords based on the London Dysmorphology Database that was enriched with entities from the “Collège Français d’Echographie Foetale” thesaurus for fetuses.

Data curation has been described in previous publications [9, 10]. The principle lies in controls at recording and regular data management. Since an RCRD/CERD cannot access the data of other RCRD/CERD, if an individual patient was seen in different centers, a new record was created, leading to duplicates. Records from the same patients but generated by different centers were mapped together using Registry Plus Linkplus [11] for the whole cohort.

Half of the accounts in the database were created for administrative staff, a quarter for non-medical non-administrative staff, and another quarter for medical staff. The task of creating a new file or updating activity was mainly done by a medical staff. Administrative staff mainly had access for activity reports, patient searches, and updating patient data.

### Focus on specific diseases and chromosomal anomalies

A focus on certain diseases was decided to show the potential information that can be obtained from CEMARA. Sub-cohorts were created for two monogenic diseases (Rubinstein Taybi and Cornelia de Lange syndromes) and two chromosomal disorders (Williams and 22q11 microdeletion syndromes). We chose these 4 conditions because they are emblematic diseases in the network, and they are commonly taken as examples to represent developmental diseases because they have been described for a very long time, are generally clinically recognizable, and are known by practitioners.

The sub-cohort of patients with chromosomal anomalies was also considered. For the latter sub-cohort, anomalies were described through chromosomal anomaly descriptor according to seven subtypes of anomalies (balanced, structural/unbalanced, autosome numerical, allosome numerical, breakage, fragile site, uniparental disomy) along with the chromosome/arm affected and the presence of mosaicism. For example, 14 subtypes were available for an unbalanced anomaly: four relating to chromosomal markers and six to duplication/deletion. The remaining referred to isochromosome, partial tetrasomy/triplication, ring chromosome, and an open subtype if none of the above applied. A few frequent anomalies had a specific code (e.g. PWS for Prader-Willi syndrome) but were still considered within the descriptor.

### Statistical method

All MDS mandatory items and chromosomal anomalies were described using frequencies for categorical data; and means ± SD or medians and interquartile range (IQR) for continuous variables according to their distribution. Some optional MDS items were only described in the subgroup analysis because only relevant in this context, such as birth parameters which are only relevant for certain diagnoses. The proportion of patients with developmental disorder in each French department was estimated as the ratio between patients seen in the RCRD/CERD for one of the targeted diseases and living in the geographic area (called “patients”) and the mean population living in the region during the study period according to the National Institute of Statistics and Economic Studies (INSEE) census (2017). The great-circle distance was used to measure distance between the residence and the place where the patients accessed care. The statistical software was R for Windows, version 3.5.1.

## Results

### Demographic information from the database

Over 10 years, data was collected for 228,243 individuals. The data included 167,361 affected patients and 60,882 unaffected patients. Unaffected patients were either healthy carriers or the relatives of an index patient, most often the parents of an affected child. The database includes vital status, and 4.8% of affected patients were reported as deceased.

The median age in affected individuals was 11 years, and the median age in non-affected individuals was 37 years. Within the affected population, 86,304 (51.6%) of patients were males and 74,319 (44.4%) were females (4% undetermined). Results are shown in Table 1. Figure 1A shows the number of patients having completed an activity record each year. The age and related sex distribution is shown in Fig. 1B.

**Table 1:** Description of the population

Number of patients in the cohort	228,243
Number of affected patients in the cohort	167,361
Median age at endpoint (in years)	
Affected	11 (Q1 = 5; Q3 = 21)
Unaffected	37 (Q1 = 30; Q3 = 43)
Male to female sex ratio	1.16
Reported death for affected patients	4.8%
Residence (Region)	
Auvergne Rhône Alpes	17,976
Bourgogne Franche Comte	7,670
Bretagne	13,701
Centre Val De Loire	5,094
Corse	562
Départements D'Outre-Mer	4,733
Grand-Est	15,179
Hauts De France	23,172
Ile De France	22,362
Normandie	2,425
Nouvelle Aquitaine	11,416
Occitanie	10,150
Pays De La Loire	12,899
Provence Alpes Côte D'Azur	13,139
Objective of visit (N = 381,209)	
Diagnosis	55%
Genetic counseling	31%
Follow-up/care	36%
Prenatal diagnosis	9.7%
Emergency	0.3%
Assertion of diagnosis	
Confirmed	34%
Likely	11%
Unlabeled	7%
Indeterminate	27%
Ongoing	19%
Missing	2%
Transmission mode (N = 73,911)	
Autosomal dominant	18,710
Autosomal recessive	7,911
Chromosomal	7,284
X-linked	4,477
Multi factorial	956
Mitochondrial	268
Unknown	34,305

	Children	Adults
Affected patients were referred by (%)		
Self	4%	16%
Patient Support Group	0%	1%
General Practitioner	2%	7%
Pediatrician	46%	17%
Other Specialist	17%	34%
Geneticist	3%	6%
Gynecologist-Obstetrician	12%	11%
Centre of maternal and child health	0%	0%
Support center	6%	3%
Multidisciplinary diagnosis centre	7%	2%
Prenatal screening centre	0%	0%
Other	1%	3%
Unknown	1%	1%

### Care pathway

A vast majority of patients were only seen once per year (82, 9%; n = 189,213), 16.5% were seen 2–3 times a year (n = 37,661), and only 0.6% more than 3 times (n = 1,369). The objectives of activity were distributed as follows: 55% were for diagnosis, 31% for genetic counseling, 36% for follow-up/care, 9.7% for prenatal diagnosis, and 0.3% for emergency. It should be noted that a patient can be seen for more than one reason (Fig. 1C). Regarding the type of visit, 85% of visits occurred on an outpatient basis (n = 310,306) and 14% were for expert medical advice for a patient hospitalized in another department or from a patient file (n = 40,601). Patients were mainly referred to a RCRD/CERD by a pediatrician when the index case was a child (46%) and by a specialist when the affected patient was an adult (34%) (Fig. 1D). 16% of adults were self-referred.

The distance to the closest expert consultation is shown in Additional file 2: Figure S1C. A median of 25.1 km was found (IQR = 6.3 km–64.2 km).

### Analysis of diagnoses

**Fig. 2:**
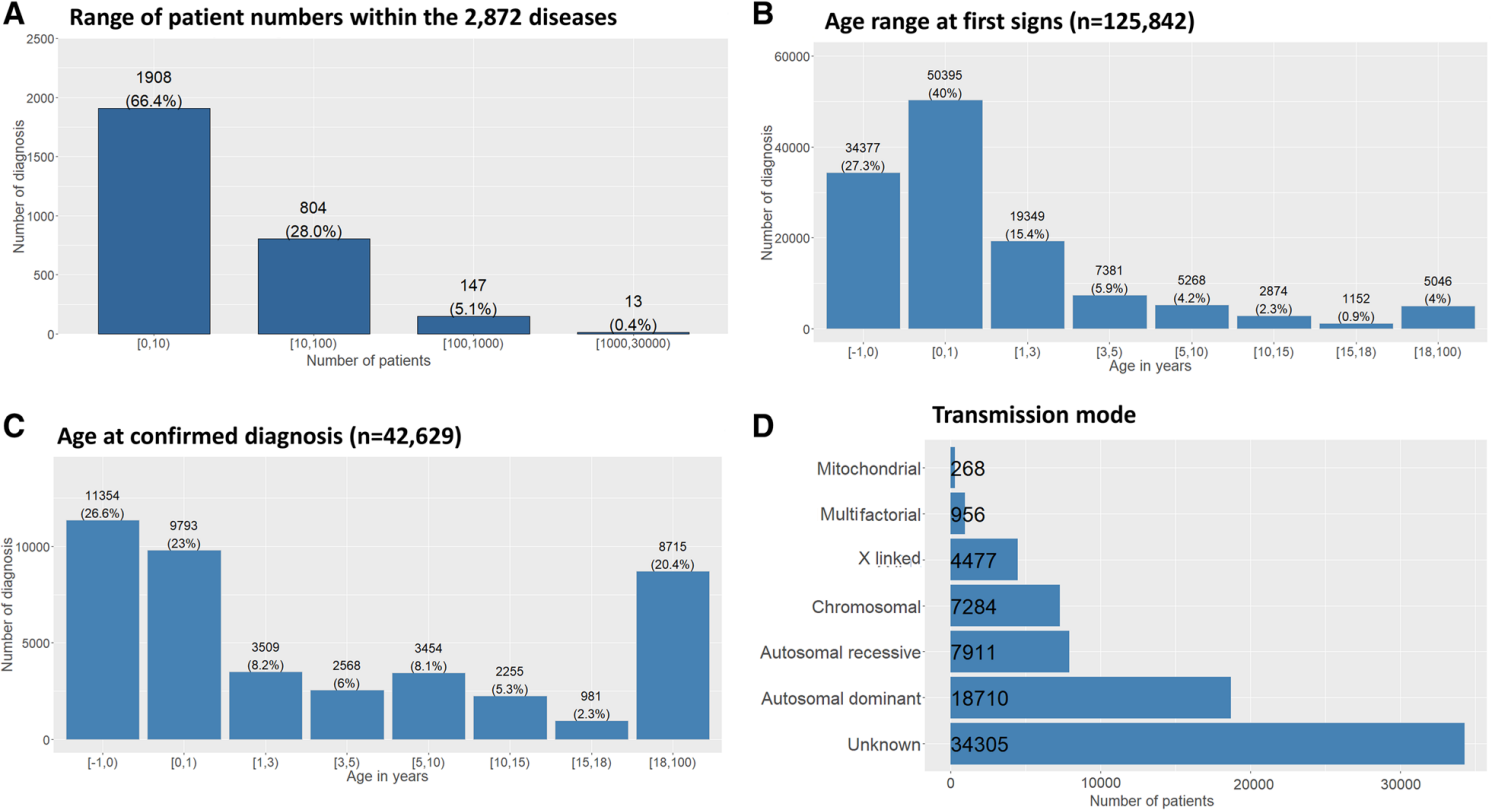
Frequency of diseases / diagnosis. **A** Range of patient numbers within the 2,872 diseases part of the AnDDI-Rares spectrum. The majority of diseases were found in 0–10 patients (66.5%, n = 1,907). 28% (n = 802) were found in 10–100 patients, 5.2% (n = 149) in 100–1,000 patients, and 0.4% (n = 11) in 1,000–10,000 patients. **B** Age range at first signs (n = 125,842). The median age at first signs is at birth (IQR = 2.5 years). The beginning of symptoms was noted within the first year of life in 67.3% of cases (n = 84,772). **C** Diagnostic status within the network. A confirmed diagnosis was found in 34% of cases (n = 56,515), undetermined in 27% (n = 10,923), ongoing in 19% of cases (n = 30,465), probable in 11% (n = 17,651), non-classifiable in 7% (n = 3,669) of patients, non-available in 2% (n = 3,669). **D** Mode of inheritance of diseases within the network. The mode of inheritance was undetermined in 46% of cases (n = 34,305), autosomal dominant in 25% of cases (n = 18,710), autosomal recessive in 11% of cases (n = 7,911), chromosomal in 10% of cases (n = 7,284), X-linked in 6% of cases (n = 4,477), suspected multifactorial in 1.3% of cases (n = 956), and mitochondrial in 0.4% of cases (n = 268). This information was optional

Among the 2,872 diagnoses in this cohort, most diseases were found in 0–10 patients (66.5%, n = 1,907), showing the frequency of ultra-RD among developmental disorders. 28% (n = 802) were found in 10–100 patients, 5.2% (n = 149) in 100–1,000 patients, and 0.4% (n = 11) in 1,000–10,000 patients (Fig. 2A). The 20 most frequent diseases are shown in Table 2, and the most frequent diagnostic categories are shown in Additional file 1: Table S2, with the proportion of patients seen within the AnDDI-Rares network versus the entire CEMARA network. Symptoms were noted prior to birth or within the first year of life in 67.3% (n = 84,772) of cases (Fig. 2B). Nearly half of patients had received no diagnosis (45.6%; n = 74,632), and the diagnostic status was unlabeled for 6.7% of patients (n = 10,923) (Table 1). When diagnosis was provided, 32% (n = 52,271) were at a disease level in Orphanet and 19.7% (n = 32,260) were in diagnostic categories. Inheritance was autosomal dominant in 25% of cases, autosomal recessive in 11% of cases, chromosomal in 10% of cases, X-linked in 6% of cases, suspected multifactorial in 1.3% of cases, and mitochondrial in 0.4% of cases (Fig. 2D). Inheritance was unknown in 46% (n = 34,305) of declared cases.

**Table 2:** The twenty most frequent diseases, with their ORPHA code, number seen in the AnDDI-Rares network, number in CEMARA (differential number seen by reference centers of other networks)

Orpha_Code	Disease	Number of patients in the_AnDDI-Rares network	Total number of patients in the CEMARA database	% of confirmed diagnosis in AnDDI-rares	% of probable diagnosis	Median age at diagnostic in months (with IQR)	
870	Trisomy 21	3512	4459	94	4	0	(6)
636	Neurofibromatosis type 1	2943	5673	68	23	36	(113)
558	Marfan Syndrome	2848	3487	33	38	180	(288)
567	22q11.2 microdélétion syndrome	2009	2592	87	6	7	(72)
908	Fragile X syndrome	1580	2339	85	5	132	(330)
648	Noonan syndrome	1374	1703	61	26	24	(128)
98249	Ehlers-Danlos syndrome	1120	1980	22	62	216	(300)
805	Tuberous sclerosis	1046	1813	69	22	12	(138)
881	Turner syndrome	993	3923	92	4	-6	(102)
666	Osteogenesis imperfeceta	766	2487	53	32	0	(36)
484	Klinefelter syndrome	762	1450	96	1	0	(246)
273	Myotonic dystrophy type 1	682	4910	84	9	276	(372)
904	Williams syndrome	681	907	86	8	21	(42)
116	Beckwith-Wiedemann syndrome	639	1090	62	23	0	(7)
3380	Trisomy18	554	618	93	4	− 6	(0)
1991	Labial cleft with or without palatine cleft	510	2374	74	14	− 6	(6)
718	Isolated Pierre Robin syndrome	412	1799	52	26	0	(0)
1906	Valproate embryofoetopathie	408	458	23	72	72	(128)
83330	Spinal muscular atrophy type 1	408	1438	82	5	276	(377)
374	Goldenhar syndrome	405	773	32	49	0	(6.5)

### Sub-cohorts analysis

**Fig. 3:**
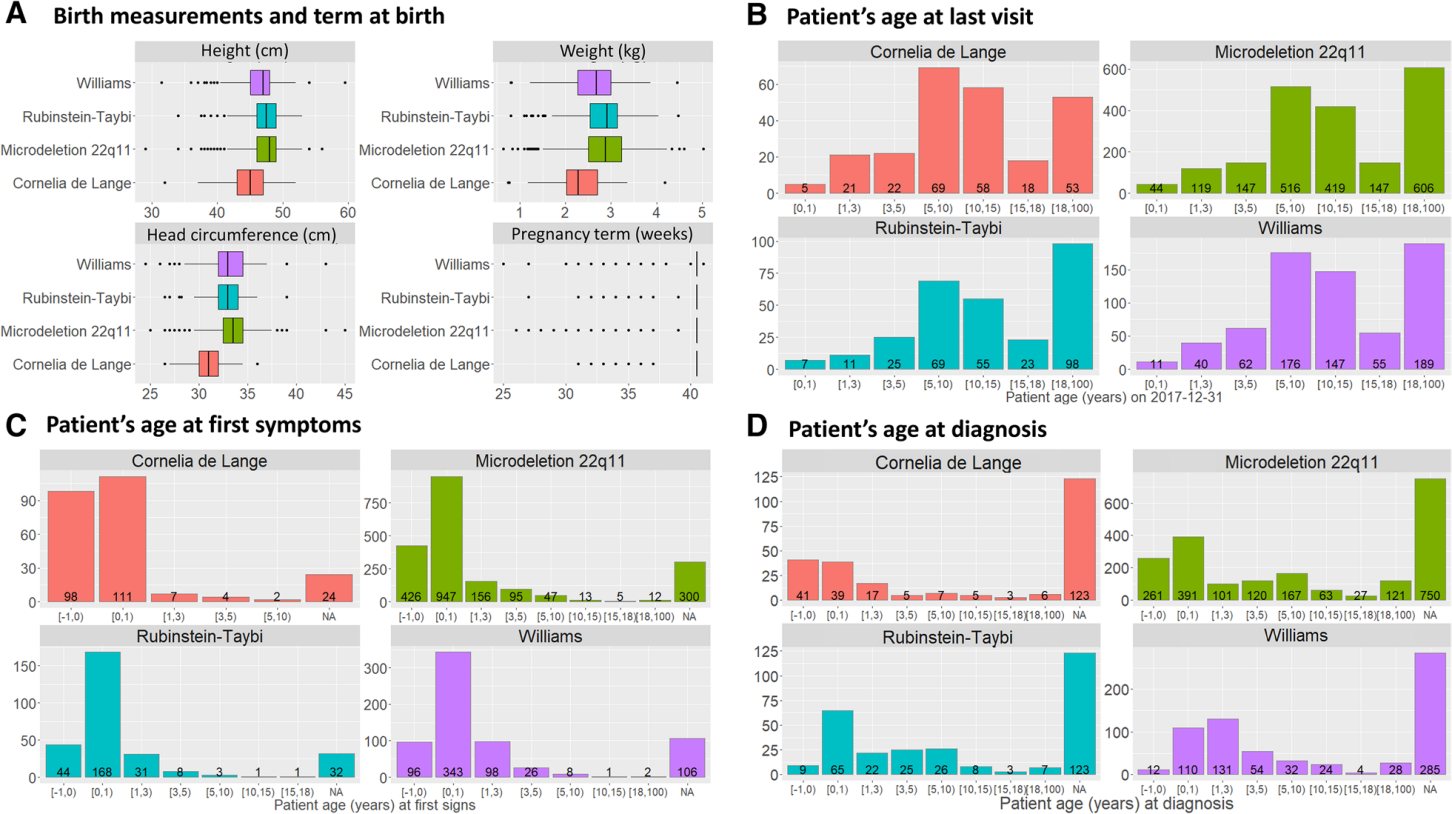
Focus on two chromosomal diseases (Williams (n = 681) and 22q11 microdeletion (n = 2,008) syndromes) and two monogenic diseases (Rubinstein-Taybi syndrome (n = 290) and Cornelia de Lange syndrome (n = 246)) as examples. **A** Birth measurements and term at birth. For each measurement by disease, number of patients. Cornelia de Lange syndrome: birth height (n = 99), birth weight (n = 110), head circumference (n = 97), term at birth (n = 252). 22q11 microdeletion syndrome: birth height (n = 764), birth weight (n = 862), head circumference (n = 710), term at birth (n = 2,033). Rubinstein Taybi syndrome: birth height (n = 171), birth weight (n = 184), head circumference (n = 150), term at birth (n = 295). Williams syndrome: birth height (n = 317), birth weight (n = 352), head circumference (n = 291), term at birth (n = 680). **B** Patient’s age at last visit. For each disease, number of patients: Cornelia de Lange syndrome (n = 246), 22q11 microdeletion syndrome (n = 1,998), Rubinstein-Taybi syndrome (n = 288), Williams syndrome (n = 680). **C** Patient’s age at first symptoms. For each disease, number of patients: Cornelia de Lange syndrome (n = 222), 22q11 microdeletion syndrome (n = 1,701), Rubinstein-Taybi syndrome (n = 256), Williams syndrome (n = 574). **D** Patients’ age at diagnosis. For each disease, number of patients: Cornelia de Lange syndrome (n = 123), 22q11 microdeletion syndrome (n = 1,251), Rubinstein-Taybi syndrome (n = 165), Williams syndrome (n = 395). On all graphs, Williams syndrome is represented in purple, 22q11 microdeletion syndrome in green, Rubinstein-Taybi syndrome in blue and Cornelia de Lange syndrome in red

The registry also allows for a more focused approach since it is possible to identify specific diseases such as Cornelia de Lange syndrome, Rubinstein syndrome, 22q11 microdeletion, and Williams’ syndrome. A manual review of double entries was conducted for these 4 sub-cohorts, on top of the previously described data curation procedures, using birthdate, sex, first name and last name. The process and results are shown in the Additional file 3: Data 1. We provide the number of patients with these four diseases found in CEMARA in Table 3, in order to provide a comparison with the biggest cohorts available in the literature. Within each disease, birth measurements and pregnancy length seemed to be consistent. Since most births occurred at term, birth measurements were congruent with the general population (Fig. 3A). Most patients were under 18 years old at their last visit, with a predominance between 5 and 15 years old (Fig. 3B). In the proportion of antenatal expression in the age at first signs, we can clearly see the effects of Cornelia de Lange syndrome in Fig. 3C. Figure 3D shows the patient age at diagnosis. The registry also provides the opportunity for short and long term outcome analysis. Among the 261 patients with Rubinstein-Taybi syndrome, one patient died at 27 days. Two of the 232 patients with Cornelia de Lange syndrome died at delivery or within hours, and two others died older than 2-year-old. Two patients among the 648 patients with Williams syndrome died before the age of 2, a third one died at 26 months. Among the 1911 patients with microdeletion 22q11.2, 17 patients died before the age of 2, 7 between 2 and 18 years, and 4 died later in life.

**Table 3:** Number of patients in CEMARA for 4 diseases taken as examples (Microdeletion 22q11.2, Williams Syndrome, Cornelia de Lange Syndrome, Rubinstein-Taybi Syndrome), and comparison with the largest cohorts published to date

CEMARA						Largest cohorts in the literature	
Orpha_id	Disease	Number of patients in AnDDI-Rares	Number of patients in CEMARA	% of confirmed diagnosis in AnDDI-Rares	% of confirmed diagnosis in CEMARA	Numbers	References
567	Microdeletion 22q11.2	1911	2592	87	88	1393	Homans et al. [20]
904	Williams Syndrome	648	907	85	86	106	Lugo et al. [21]
199	Cornelia de Lange Syndrome	232	360	50	52	486	Mehta et al. [22]
783	Rubinstein-Taybi Syndrome	261	373	73	73	93	Schorry et al. [23]

Additional file 1: Figure S1C shows the distance travelled by the families to go to a reference center for their disease. For example, patients with Rubinstein-Taybi syndrome often travel to Bordeaux, which is home to the expert center for this disease.

**Fig. 4:**
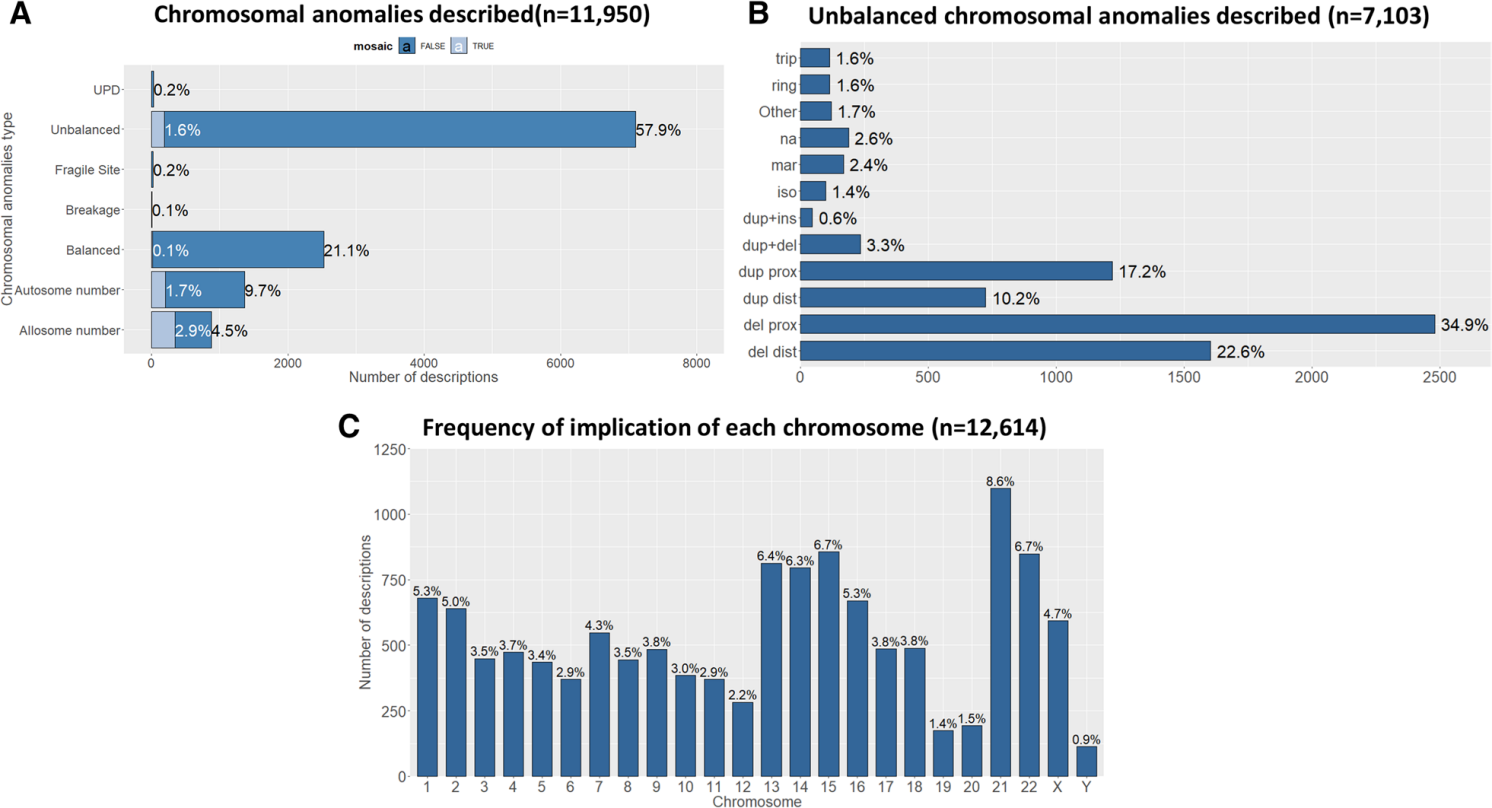
Focus on chromosomal anomalies. **A** Chromosomal anomalies described (n = 11,950). The unbalanced structural anomalies are mostly described (59.4%, n = 7103) whereas number anomalies only represent 18.8% (n = 2247). **B** Unbalanced chromosomal anomalies described (n = 7,103). Distal deletion: 22.6% (n = 1603); proximal deletion: 34.9% (n = 2480); distal duplication: 10.2% (n = 724); proximal duplication 17.1% (n = 1218); duplication and deletion (only one chromosome): 3.3% (n = 234); proximal duplication (inserted elsewhere): 0.6% (n = 46); isochromosome: 1.4% (n = 98); Marker: 2.4% (n = 167); Missing data: 2.6% (n = 187); Other: 1.7% (n = 119); ring chromosome: 1.6%(n = 114); partial triplication/tetrasomy: 1.6% (n = 113).**C** Frequency of implication of each chromosome (n = 12,614). Chromosomal anomalies are more frequent for all acrocentric chromosomes as compared to non-acrocentric chromosomes, the more represented being chromosome 21 (n = 1,086; 8.6%), chromosome 15 (n = 856; 6.7%), and chromosome 22 (n = 849; 6.7%)

**Fig. 5:**
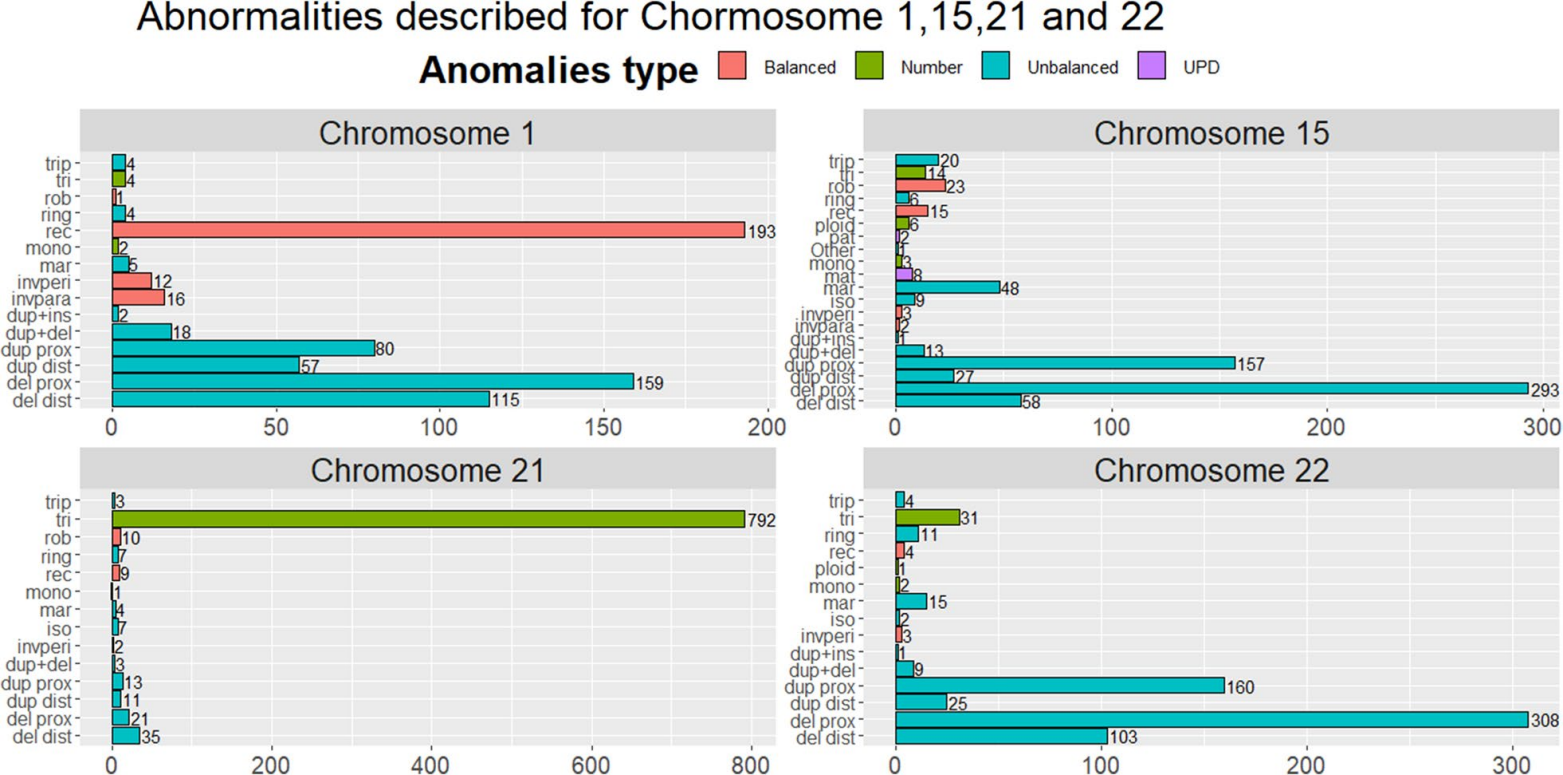
Distribution of chromosomal anomaly for the 3 most frequent chromosomes implicated, as well as chromosome 1, as an example of non-acrocentric chromosome. For these graphs, "del dist": distal deletion; "del prox": proximal deletion; "dup dist": distal duplication; "dup prox": proximal duplication on the same chromosome; "dup + del": duplication and deletion (only one chromosome); "dup + ins": proximal duplication (inserted elsewhere); "invpara": paracentric inversion; "invperi": pericentric inversion; "iso": isochromosome; "mar": Marker; "mat": maternal; "mono": monosomy; "pat": paternal;"ploid": triploidy/tetraploidy; "rec": reciprocal translocation; "ring": ring chromosome; "rob": robertsonian translocation; "tri": trisomy; "trip": partial triplication/tetrasomy; "Other": other anomalies. **A** Chromosome 1, reciprocal translocation was the most frequent chromosomal anomaly, followed by proximal and distal deletions. **B** Chromosome 15, proximal deletions or duplications were the most frequent chromosomal anomalies. **C** Chromosome 21, the trisomy of the whole chromosome was the most frequent chromosomal anomaly. **D** Chromosome 22, proximal deletions or duplications were the most frequent chromosomal anomalies

The CEMARA registry includes 34,737 patients carrying chromosomal anomalies, among which 22,019 are part of the AnDDI-Rares network. Figure 4A shows the repartition of chromosomal anomalies and especially for unbalanced anomalies (Fig. 4B). Figure 4C showed the frequency at which each chromosome is implicated. Chromosomal anomalies were more frequent for all acrocentric chromosomes as compared to non-acrocentric chromosomes. The most frequently affected chromosomes were 21 (8.6%), 15 (6.7%), and 22 (6.7%). It was then possible to focus on each chromosome. Figure 5 represents the distribution of chromosomal anomalies for the 3 most frequent chromosomes implicated, but also chromosome 1 as an example of non-acrocentric chromosome. In chromosome 1, reciprocal translocation was the most frequent chromosomal anomaly, followed by proximal and distal deletions. For chromosome 15 and 22, proximal deletions or duplications were the most frequent chromosomal anomalies. For chromosome 21, the trisomy of the whole chromosome was the most frequent chromosomal anomaly.

## Discussion

We present here the organization of the registry for rare developmental disorders, including intellectual disability or not as part of the AnDDI-Rares network, and provide an analysis of what we have learned from the first 10 years using the CEMARA database.

Information and knowledge about RD is usually the result of data collection and registries implemented with academic and/or commercial interests and with a limited scope. Interestingly, the online rare disease database Orphanet [12] indexes a total of more than 700 registries and databases on RD involving European research, and it can thus be used to estimate disease prevalence [13]. These registries and databases have a variety of aims and differ in their organization, quality and database structure, usually monitoring one disease or a group of related diseases [14–16]. In order to encourage the development of knowledge about RD, several countries have launched national initiatives to build registries including all RDs and with the suggestion of international cooperation, in particular within the European Reference Networks [17]. These registries, if properly implemented with accurate and high-quality clinical data and long-term support, can facilitate health service planning, epidemiological research and clinical trial recruitment. Nevertheless, the collected data must be congruent with the aims of the registry. Registries are particularly important for rare or poorly-understood diseases that affect small numbers of patients, complex delayed diagnoses, a propensity for variable standards of care and limited treatment options.

The first French RD initiative, CEMARA, has been collecting information on RD epidemiology and related medical activities from RCRD and CERD on a national-level since 2007. To date, the data entered in CEMARA has already been used by some networks for RD [18]. Other publications have also been facilitated by CEMARA’s infrastructure [19]. More specifically, data on age, sex ratio, type of care, median distances travelled by patients, the most frequent type of referrals, and diagnosis categories or precise diagnoses (when available) can be obtained.

Interestingly, when compared with the data obtained for the Head and Neck Network, in which nearly 80% of patients are required to visit Paris hospitals to obtain diagnosis, care or follow up [18], the distribution of RCRD/CERD within the AnDDI-Rares network (Additional file 2: Figure S1A) has optimized the distance patients must travel to obtain specialist care for RD.

Unlike most registries collecting detailed information on specific rare diseases, the main aim of this nationwide database is not to improve knowledge on the natural history of diseases. This is because the scope of CEMARA covers all rare diseases, which is too vast to achieve such a goal. Even so, some aspects of a disease’s natural history can be analyzed straightforwardly (age at first signs, age at death, e.g.) while others can be inferred (based on the care pathway, age at diagnosis, e.g.). A deep phenotypic description is possible through HPO terminology, but we observed that not all physicians extensively code this optional information.

In addition, we demonstrated the information that can collected on specific topics using the examples of four well-known easily recognizable diseases, including two chromosomal abnormalities (22q11 microdeletion and Williams syndromes) and two mendelian diseases (Cornelia de Lange (CdLS) and Rubinstein-Taybi syndromes (RTS)). In these examples, the large number of patients in the database makes it possible to compare with other initiatives [20–23]. CEMARA collects a greater number of patients compared with the the biggest available national cohort for three of the four diseases chosen herein. Only the Children’s Hospital of Philadephia’s Cohort on Cornelia de Lange was bigger than CEMARA’s.

In the field of RD, patient organizations are usually the best resource for reaching out to a significant number of patients for a given disease. However, this national database proves to be an even more efficient tool to collect patient data since it includes information on every patient seen in an RCRD and some CERD. It can thus provide larger cohorts than those currently found in the literature for most RD. Similarly, over time the database has accumulated a vast number of patients with the various chromosomal abnormalities, diagnosed by karyotype, FISH or array-CGH. Data on chromosomal abnormalities associated with developmental phenotypes can be of great interest, yet there are no extensive epidemiological references in the literature since the use of chromosomal microarrays became more common. Researchers can now solicit the network if they want to focus on a certain disease and contact the referring clinicians all over the country for additional information. This possibility will be interesting for international collaborations on the increasing numbers of ultra-RD, but also for long-term follow-up of well-known diseases.

The CEMARA registry is comparable with other national projects that have been published in the literature. In Europe, CEMARA resembles most its Italian counterpart, which launched in 2001 as a government baseline project to support health policy decision-making in the field of RD [24, 25]. They established a national registry of RD as a network of regional networks through 247 formally designated centers with recognized expertise, reaching full coverage of the country by 2011. After a common data set was defined for the country, they performed different quality control processes at regional and national levels. One of the main issues was tracking duplicate records. Up to June 2012, they recorded 110,841 patients. Data was carefully monitored through a validation process using formal criteria, and issues in the data were corrected by the data sources. Data of age at onset and sex distribution were provided for about 400 diseases, and incidence and/or birth prevalence provided for 275 diseases and 47 disease groups, which, altogether, comprise a substantial part of the known RD. The main difference lay in the fact that CEMARA was launched as a national project, allowing a nation-wide common data collection from the outset, thus a greater hindsight, compared with the Italian project. Both projects shared similarities regarding the type of data which may foreshadow comparative and/or pooling data studies.

Other initiatives exist outside of Europe. In the majority of cases, the strategy was to create alliances of existing RD registries, with the creation of a central repository aiming to improve consistency, harmonize data, support the development of knowledge on RD, share data, enhance research collaboration, improve interoperability, and reduce costs. The USA National Institute of Health launched a movement to create a Global RD Patient Registry and Data Repository in 2010 [26], but unlike CEMARA the contribution to this RD-hub was based on goodwill. In China, a nationwide RD registry has been set up along with a bio-bank of genomic data to provide standardization and create research collaborations, both domestic and international [27, 28]. In 2017, Japan decided to combine data from 300 RD projects through a cross-sectional data integration platform (RADDAR-J) [29], aiming to promote data sharing and secondary use for research and collaboration. This Japanese initiative only focused on 300 RD, thus lacked information compared to CEMARA. A global observatory for rare disease could be achieved through the combination of these various initiatives, to the great benefit of patients: given the small number of cases in each country, it is of paramount importance that data be analyzed on the widest possible scope.

This work provides elements relative to the functioning of the database over the first 10 years. We have identified many important limitations that we wish to share with other countries which are attempting to implement nationwide epidemiological projects. Epidemiological information regarding RD is challenging to collect for a number of reasons, including the coding and classification of RD. In our case, this difficulty was overcome with the implementation of a unique disease identifier resulting from the exhaustive work of the online rare disease database Orphanet on the labeling of diseases: OrphaCodes. While exhaustiveness is usually is a challenge for any registry, public funding conditional to participation in the CEMARA project will remain a significant incentive. Unfortunately, such an incentive is not in place for CERD, even if the French Ministry of Health is providing other operational support in order to facilitate inclusion. Indeed, the RCRD have an obligation to enter all of their activity into the CEMARA database to keep their funding, unlike the CERD. As a result, most CERD do not collect patient data, so there are limits to the epidemiological work that can be carried out. Another minor limitation is that of duplicates: patients can consult in different RCRD/CERD, which implies the creation of a new file, and so any multicenter analysis requires the identification of potential duplicates. Another limit is a lack of homogeneity in the way data are entered in the different RCRD/CERD since the definition of items may not always be straightforward. For instance, a physician may consider a diagnosis as confirmed based on clinical evidence, while another may consider that confirmation is achieved only after genetic confirmation. Some improvements have been made to overcome these issues, including a frame of reference to homogenize the way data is entered, and communication in meetings to insist on the importance of epidemiology in France. A major issue is the surveillance of patients with no diagnosis, which is considered a priority of the third national RD plan [30]. Indeed, the database does not permit to identify age at clinical diagnosis, age at diagnosis of a category, or a precise clinical diagnosis by a chromosomal/molecular confirmation. This issue will be improved in the next version of the database, since it is part of the vast epidemiological national surveillance project for undiagnosed patients. It is also difficult to ensure that the data are updated when a diagnosis is made, particularly when the results are not delivered in the context of a novel referral to the RCRD/CERD. Despite the limited amount of information collected, specific studies could be performed within the network through the identification of the exact number of patients by RCRD/CERD in France with a disease of interest. This would enable national studies to be performed, or, through linkage with other sources, to seek data that could be used to improve the management of RD, facilitate research, such as phenotype/genotype correlations or drug surveillance, or exposes economic issues such as the burden of RD. New perspectives are currently raising with the launch of a registry of patients with no diagnosis, enabling to better identify patients without diagnosis, to whom new research programs could be proposed. Also, at the dawn of the arrival of therapeutic projects in RD, the database will allow the selection of potential candidates for a therapeutic trial according to their demographic characteristics. For this purpose, although individual sites cannot access data from other sites, it is possible to ask the project coordination team, in agreement with the network, for the number of people affected by selected criteria and their referring center. In this way, the applicant can contact his or her colleagues in the framework of his project.

## Conclusions

Thanks to the national epidemiological project launched by the French Ministry of Health 10 years ago, the main characteristics of French patients with RD are available, potentially leading to the identification of patients for specific studies. Issues with exhaustiveness shall be progressively resolved thanks to continuous human and financial support, and coding methods are continuously improved through harmonization work. AnDDI-Rares’ experience with CEMARA will benefit other French rare disease networks since they are all joining the French National Rare Disease Registry, a registry integrating all CEMARA data that is to be deployed more broadly throughout the national network of expert centers. New perspectives are also being developed with the expansion of MDS data collection to all rare disease networks in France and Europe.

## Supplementary Information

**Additional file 1: Table S1.** The minimum data set of the CEMARA database. **Table S2.** The twenty most frequent groups of diseases, with their ORPHA code, number seen in the AnDDI-Rares network, number in CEMARA (differential number seen by reference centers of other networks)

**Additional file 2: Figure S1.** Access to care and referrals. A: Map of the network: RCRD (round)/CERD (triangles) of the network, with inactive centers for entering patients in the CEMARA database in yellow. B: Number of patients with a developmental disorder referred to an AnDDI-Rares RCRD/CERD varied according to the French departments. The map should be analyzed along with supplementary figure 1A. C: Distance necessary to access to an expert consultation in the total population, and for the four diseases of interest (Rubinstein-Taybi, Cornelia de Lange, 22q11 and Williams syndromes). A median of 25.1 km was found (Q1: 6.3 km – Q3: 64.2 km) for the total population

**Additional file 3: Data 1.** Handling of duplicates. For the 4 diseases of interest, LinkPlus detected 247 potential pairs including 163 exact match pairs. For the remaining pairs (84), we conducted a manual review. The file with the last activity was kept. It resulted that most of them were not duplicates (58). Finally, we pooled all pairs of duplicates and observed triples (8). As a result, we excluded the 204 duplicate files from analysis

## Data Availability

Please contact author for data requests.
