# Supplementary material for: 10 years of CEMARA database in the AnDDI-Rares network: a unique resource facilitating research and epidemiology in developmental disorders in France
Source: Orphanet J Rare Dis. 2021 Aug 4;16:345. doi: 10.1186/s13023-021-01957-4 (PMC8335940; doi:10.1186/s13023-021-01957-4)
Supplement: Supplementary file 1 — Additional file 1: Table S1. The minimum data set of the CEMARA database. Table S2. The twenty most frequent groups of diseases, with their ORPHA code, number seen in the AnDDI-Rares network, number in CEMARA (differential number seen by reference centers of other networks) [file 13023_2021_1957_MOESM1_ESM.docx]

Supplementary table 1: The minimum data set of the CEMARA database

|  | Item |
| --- | --- |
| 1.1 | Is a fetus? |
| 1.2 | First name |
| 1.3 | Surname |
| 1.4 | Married name* |
| 1.5 | Gender |
| 1.6 | Birth date |
| 1.7 | Uncertain birth date |
| 1.8 | City of birth code |
| 1.9 | Country of birth |
| 1.10 | Country of residence |
| 1.11 | City of residence |
| 1.12 | Patient status |
| 1.13 | Healthy carrier |
| 1.14 | Relationship with the proband |
| 1.15 | Comment for Relationship with the proband |
| 1.16 | Is deceased |
| 1.17 | Date of death |
| 1.18 | Type of pregnancy termination |
| 1.19 | Is fetopathology performed ? |
| 1.20 | Laboratory and responsible physician for fetopathology |
| 2.1 | Who/which facility sent the patient ? |
| 2.2 | Assessment of the diagnosis at center admission |
| 2.3 | Rare Disease Care Unit Name |
| 2.4 | Primary physician |
| 3.1 | Type of personnel performing the declared activity. |
| 3.2 | Comment on Other type of professional |
| 3.3 | Name of the professional performing the activity. |
| 3.4 | Date on which the declared patient care was performed |
| 3.5 | Place of consultation |
| 3.6 | Care type |
| 3.7 | Context of care |
| 4.1 | Moment at which symptoms first appeared? |
| 4.2 | Age at onset (in months) |
| 4.3 | Period of diagnosis* |
| 4.4 | Age at diagnosis in months* |
| 4.5 | Diagnosis |
| 4.6 | Current state of the diagnosis |
| 4.7 | Confirmation Mode |
| 4.8 | Death due to the rare disease |
| 4.9 | Is the case isolated or familial at the time of observation (assessed by the RD professional)? |
| 4.10 | Detailed transmission mode* |
| 4.11 | GenAtlas code* |
| 4.12 | Mutation* |
| 4.13 | Comment on diagnosis |
| 4.14 | Keywords (LDDB)* |
| 5.1 | Abnormality diagnosed prenatally* |
| 5.2 | Medically assisted procreation* |
| 5.3 | Offer of abortion for medical reasons* |
| 5.4 | Abnormality diagnosed prenatally (how many weeks)* |
| 5.5 | Does the patient have a prenatal malformation?* |
| 5.6 | Was the patient born at full-term? If not clarify the term.* |
| 5.7 | Height at birth* |
| 5.8 | Weight at birth* |
| 5.9 | Head circumference at birth* |
| 5.10 | Patient born from a relationship between related parties |
| 5.11 | Patient in a relationship with a related person |
| 5.12 | Examination performed* |
| 5.13 | Is anomaly detected through examination ?* |

*: optional

Supplementary Table 2: The twenty most frequent groups of diseases, with their ORPHA code, number seen in the AnDDI-Rares network, number in CEMARA (differential number seen by reference centers of other networks)

| Orpha_Code | Group of disease label | Number of patients in the_AnDDI-Rares network | Total number of patients in the CEMARA database | Median age at first signs in months (with IQR) | |
| --- | --- | --- | --- | --- | --- |
| 102369 | Syndromic ID | 22656 | 26424 | 0 | (18) |
| 68335 | Chromosomal anomaly | 22019 | 34737 | 0 | (9) |
| 1685 | Rare non syndromic ID | 7543 | 10071 | 24 | (26) |
| 102285 | Malformative syndrome without ID | 4219 | 4538 | 0 | (6) |
| 106 | Autism | 2891 | 5685 | 24 | (12) |
| 217598 | Non malformative neurologic and psychiatric disease | 1580 | 2525 | 20.5 | (18) |
| 98036 | Ear Nose and Throat disease | 808 | 1270 | 25.5 | (72) |
| 98047 | Rare infertility | 706 | 1285 | 372 | (84) |
| 183570 | Malformative syndrome with short stature | 703 | 774 | 0 | (12) |
| 88991 | Congenital heart malformation | 703 | 777 | -6* | (6) |
| 166463 | Syndrome with epilepsy | 693 | 3470 | 6 | (27) |
| 68341 | Multiple congenital anomaly with dysmorphism | 601 | 643 | 0 | (6) |
| 90692 | Rare endocrine disease with growth anomaly | 534 | 952 | 6 | (48) |
| 68378 | Limb congenital anomaly | 459 | 515 | 0 | (6) |
| 90642 | Syndromic deafness of genetic origin | 426 | 640 | 18 | (60) |
| 139024 | Syndromic obesity or overgrowth | 409 | 441 | 24 | (41) |
| 98044 | Central nervous system malformation | 408 | 545 | -6* | (6) |
| 87277 | Rare ID | 368 | 1803 | 12 | (23) |
| 98006 | Rare neurologic disease | 289 | 3293 | 0 | (12) |
| 93545 | Malformation of kidney and/or urinary tract | 268 | 362 | -6* | (0) |

*: -6 means detected at an antenatal stage
