# Supplementary figures and images for: 10 years of CEMARA database in the AnDDI-Rares network: a unique resource facilitating research and epidemiology in developmental disorders in France
Source: Orphanet J Rare Dis. 2021 Aug 4;16:345. doi: 10.1186/s13023-021-01957-4 (PMC8335940; doi:10.1186/s13023-021-01957-4)

## Slide 1
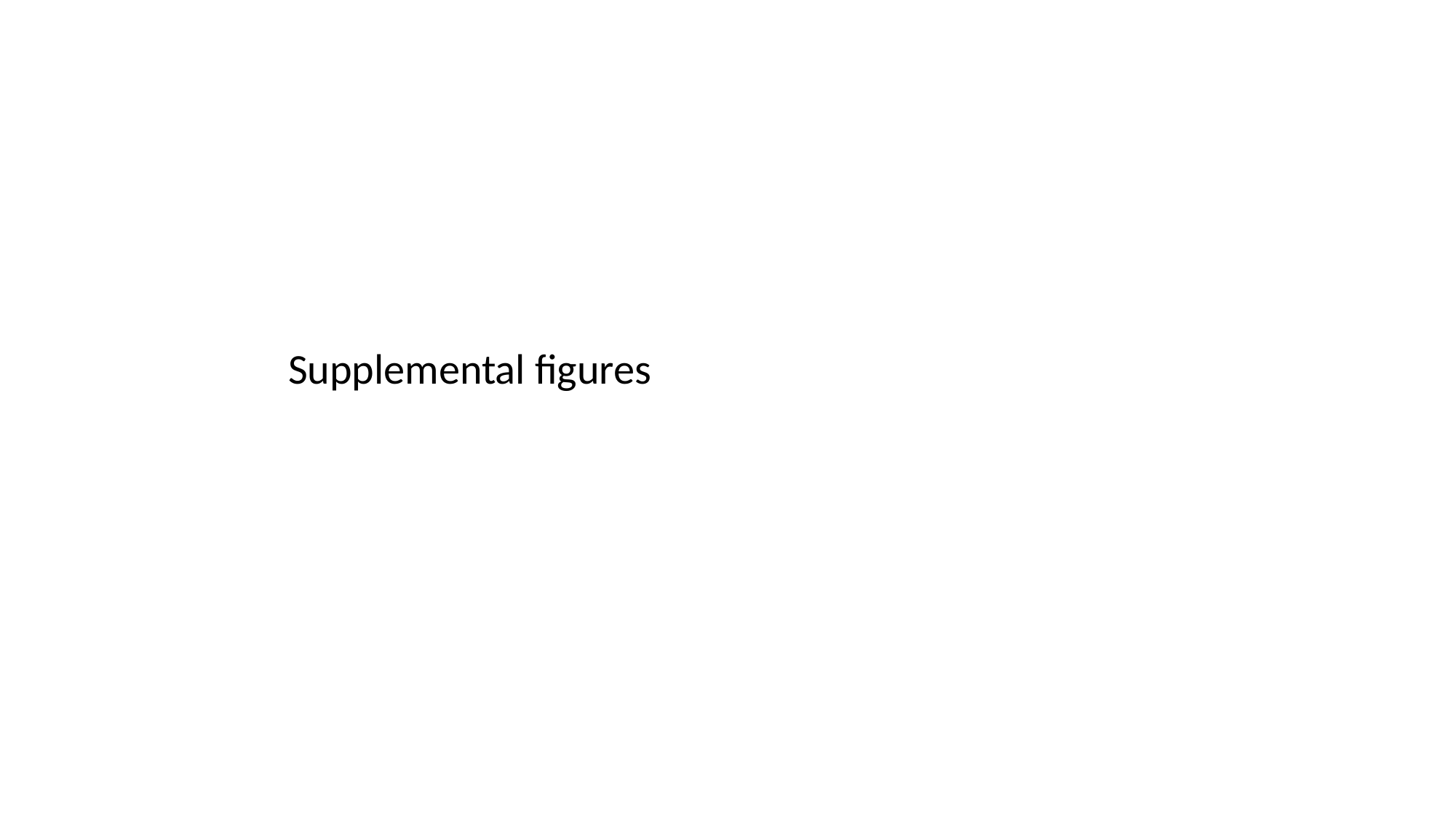

Supplemental figures

## Slide 2
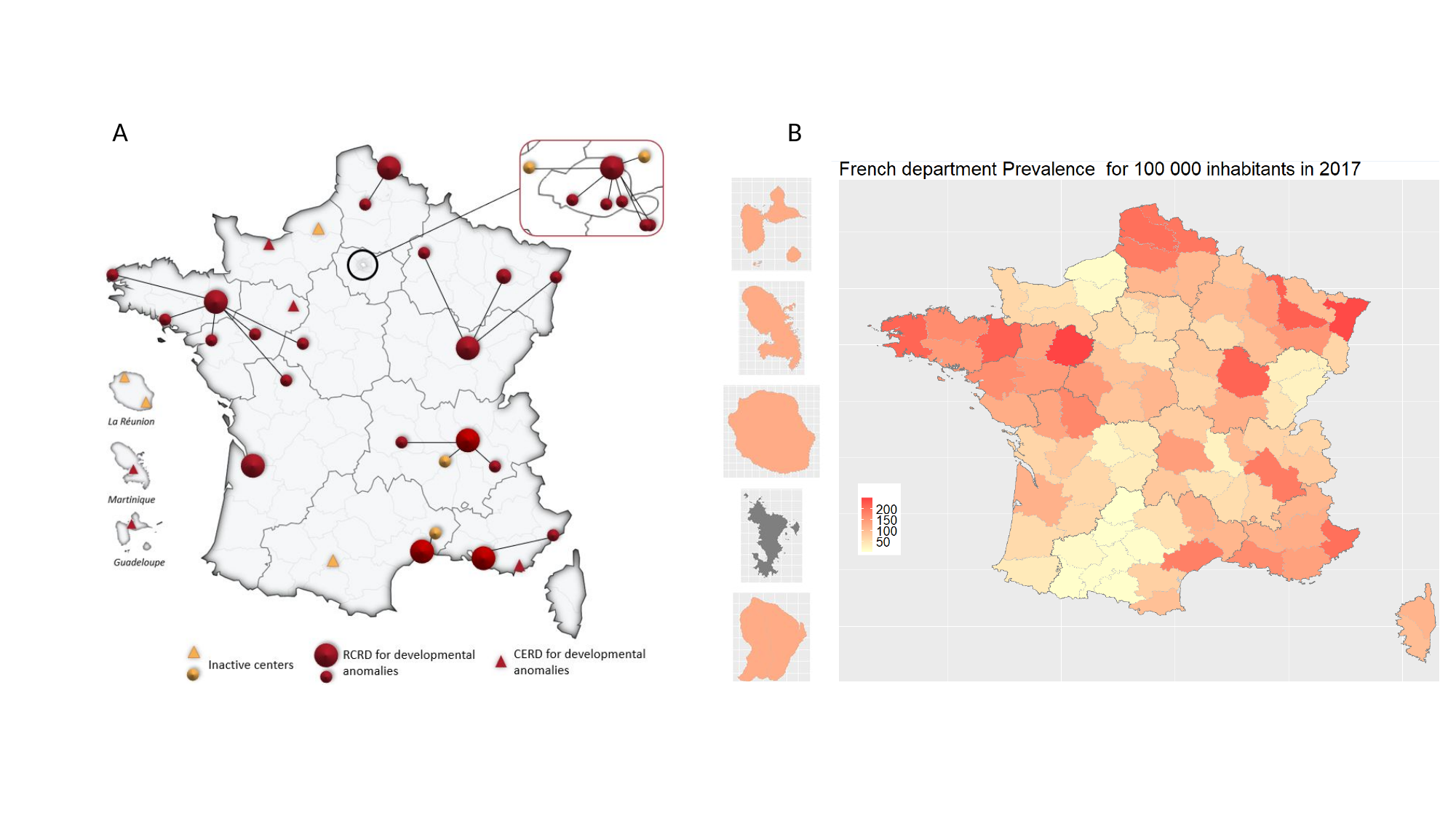

A
B

## Slide 3
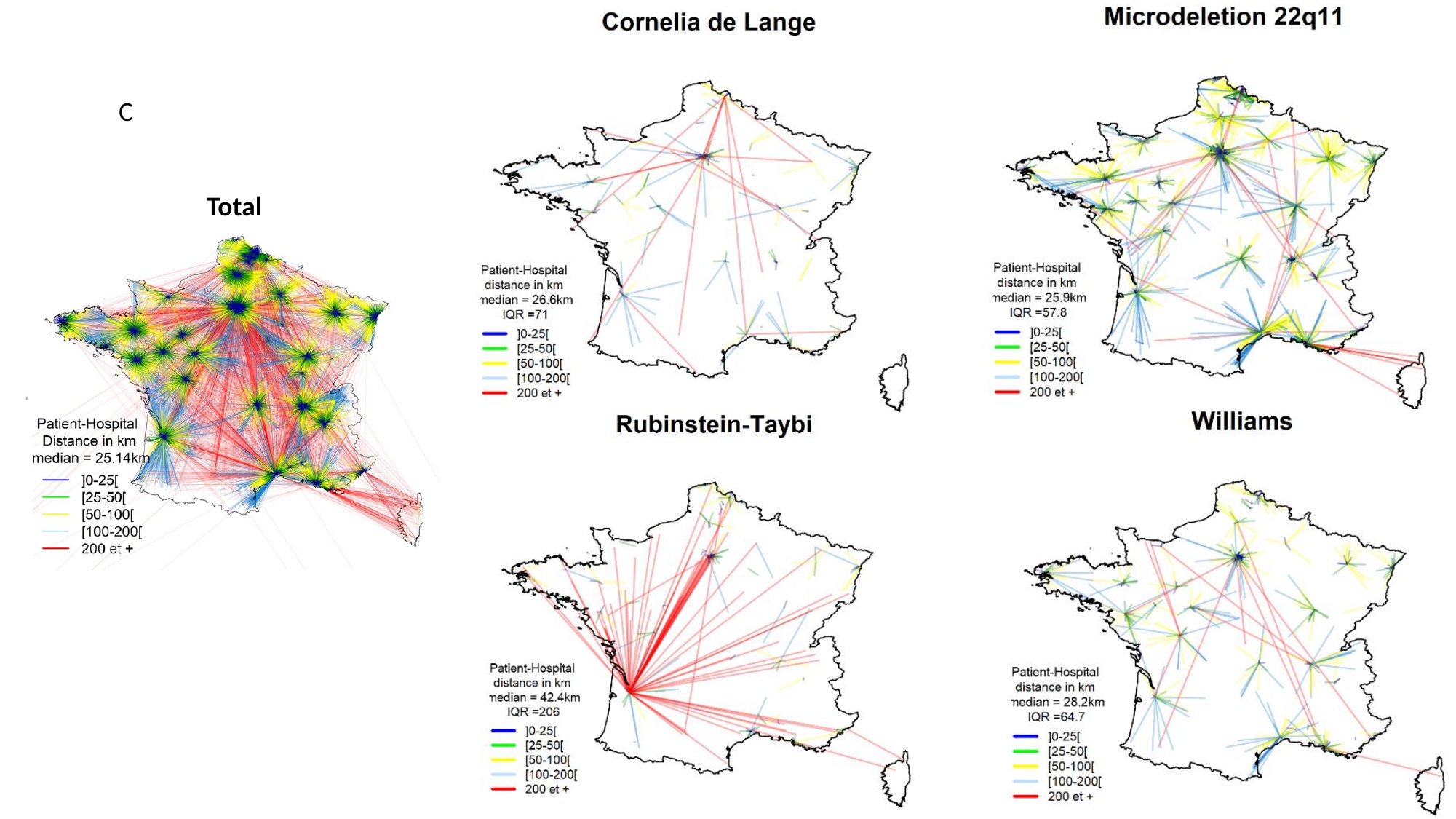

C
Total

Supplement: Supplementary file 2 — Additional file 2: Figure S1. Access to care and referrals. A: Map of the network: RCRD (round)/CERD (triangles) of the network, with inactive centers for entering patients in the CEMARA database in yellow. B: Number of patients with a developmental disorder referred to an AnDDI-Rares RCRD/CERD varied according to the French departments. The map should be analyzed along with supplementary figure 1A. C: Distance necessary to access to an expert consultation in the total population, and for the four diseases of interest (Rubinstein-Taybi, Cornelia de Lange, 22q11 and Williams syndromes). A median of 25.1 km was found (Q1: 6.3 km – Q3: 64.2 km) for the total population [file 13023_2021_1957_MOESM2_ESM.pptx]
