# Supplementary material for: 10 years of CEMARA database in the AnDDI-Rares network: a unique resource facilitating research and epidemiology in developmental disorders in France
Source: Orphanet J Rare Dis. 2021 Aug 4;16:345. doi: 10.1186/s13023-021-01957-4 (PMC8335940; doi:10.1186/s13023-021-01957-4)
Supplement: Supplementary file 3 — Additional file 3: Data 1. Handling of duplicates. For the 4 diseases of interest, LinkPlus detected 247 potential pairs including 163 exact match pairs. For the remaining pairs (84), we conducted a manual review. The file with the last activity was kept. It resulted that most of them were not duplicates (58). Finally, we pooled all pairs of duplicates and observed triples (8). As a result, we excluded the 204 duplicate files from analysis [file 13023_2021_1957_MOESM3_ESM.docx]

**Supplementary Data 1: Handling of duplicates**

For the 4 diseases of interest, LinkPlus [11] detected 247 potential pairs including 163 exact match pairs. For the remaining pairs (84), we conducted a manual review. The file with the last activity was kept. It resulted that most of them were not duplicates (58). Finally, we pooled all pairs of duplicates and observed triples (8). As a result, we excluded the 204 duplicate files from analysis.
